# Evaluation of Nanofiltration Membranes for the Purification of Monosaccharides: Influence of pH, Temperature, and Sulfates on the Solute Retention and Fouling

**DOI:** 10.3390/membranes12121210

**Published:** 2022-11-30

**Authors:** Buddhika Rathnayake, Hanna Valkama, Markku Ohenoja, Jasmiina Haverinen, Riitta L. Keiski

**Affiliations:** 1 Environmental and Chemical Engineering Research Unit, Faculty of Technology, University of Oulu, P.O. Box 4300, FI-90014 Oulu, Finland; 2 Unit of Measurement Technology, Kajaani University Consortium, University of Oulu, P.O. Box 127, FI-87400 Kajaani, Finland

**Keywords:** nanofiltration, lignocellulosic biomass, monosaccharide recovery, negative retention, fouling, diafiltration, design of experiments

## Abstract

Furfural, acetic acid, and sulfates are found in the hemicellulose (HMC) fraction of lignocellulosic biomass. Separation of furfural, acetic acid, and sulfates from monosaccharides by four nanofiltration (NF) membranes was evaluated with a model solution of glucose, xylose, furfural, acetic acid, and sulfates. Results showed that Alfa Laval NF99HF is the most promising membrane to purify monosaccharides, with the retentions of xylose (85%), glucose (95%), and with the minimum sulfate retention. pH has the highest impact on the retention of all solutes and there is no significant effect of temperature on the retentions of sulphates and acetic acid. Lower pH and temperature are favored to maximize the monosaccharide retention and to remove acetic acid while retaining more furfural with the monosaccharides. Moreover, fouling tendency is maximized at lower pH and higher temperatures. According to the statistical analysis, the retentions of glucose, xylose, furfural, sulfates, and acetic acid are 95%, 90%, 20%, 88%, and 0%, respectively at pH 3 and 25 °C. The presence of sulfates favors the separation of acetic acid and furfural from monosaccharides.

## 1. Introduction

Lignocellulosic biomass is one of the abundant and renewable resources for the production of biofuels and chemicals [1,2]. In biorefineries, lignocellulosic raw materials typically undergo various pre-treatment processes including chemical, physical, physicochemical, and biological processes to fractionate them into cellulose, hemicellulose (HMC), and lignin [3]. HMC fraction is a diverse group of polysaccharides, which consists of various monosaccharide units such as glucose, xylose, galactose, arabinose, and mannose [4,5]. Consequently, HMCs have a higher water solubility due to their branched structure [6]. In addition to the monosaccharide units, the HMC fraction contains valuable byproducts e.g., furfural, hydroxymethylfurfural (HMF), and impurities e.g., acetic acid, formic acid, aromatic compounds, extractives, and inorganic residues [7,8].

Separation of HMC sugars from byproducts and impurities is one of the major challenges in the upstream processing in biorefineries. For the efficient upstream processing of HMC streams, one of the key strategies is developing membrane technologies as low-energy solutions to remove soluble non sugar components and concurrently to achieve higher concentrations and ensure higher purity of the HMC sugars [9]. The recovery of valuable compounds could improve the efficiency of the downstream processes but also propose new sources of those wanted compounds.

Different methods have been reported in the literature for the purification of monosaccharides in lignocellulosic biomass. Huang et al. [10] reviewed the separation technologies used in biorefineries and stated that the most common methods, distillation, and evaporation require higher energy than the membrane separation, which has been studied in the recent research. Thus, pressure driven membrane technologies are more promising pre-treatment methods in biomass-based production processes [11].

Simultaneous removal of impurities and concentration of HMC have occasionally been reported in the literature. Instead, single compound separation has been studied with membranes more frequently. Restolho et al. [12] found that Alfa Laval RO99 rejects 96% and 93% of glucose and xylose, respectively, with single compound streams. Qi et al. [13] investigated the separation of furfural from monosaccharides and showed that rejections decreased with increasing pH and temperature for NF90 and NF270 membranes.

Negative retention, where the solute ions are enriched in the permeate compared with the bulk feed, can be seen in certain circumstances especially in nanofiltration (NF) of organic solutions [14,15,16]. In water purification applications, where the permeation of only water is beneficial, the negative retention is harmful. However, in the removal of impurities from solutions, the negative retention of impurities would be beneficial. Schlackl et al. [17] reported that negative retention of sulfates containing biomass hydrolysates occurs due to intermolecular interactions in the feed.

Membrane fouling is one of the major issues in membrane separation processes that causes the reduction in the permeate flux and affects the quality of the filtered products. Several researchers have highlighted the fouling phenomenon in their studies [3,18,19,20]. Gönder et al. [19] reported reduced membrane fouling at the alkaline condition (pH 10) and at low temperature (25 °C) when they investigated the biologically treated wastewater by NF. Comprehensive fouling studies to obtain experimental values for the individual fouling terms have been performed in e.g., purification of HMC streams [3,18] and desalination of brackish water [20], using resistance-in-series model.

To find more information on the effect of pH, temperature and sulfates on solute retentions and fouling, these phenomena were explicitly studied with NF membranes using a model solution containing glucose, xylose, furfural, acetic acid, and sulfates. The novelty of our work lies with the selected complex multi-compound stream which contains sulfates and with the systematic study of the effects on solute retention and fouling. Findings can be utilized to facilitate the efficiency of the biorefineries by intensifying the reclamation of valuable components from the side streams by the means of membrane technology.

## 2. Materials and Methods

### 2.1. Model Solution

The model solution was chosen based on the literature [21,22,23] and the typical concentrations of selected compounds in filtrates after lignin precipitation from the organosolv process at the Fraunhofer CBP. The solution was prepared by adding a pre-determined amount of chemicals into ultrapure water produced by a Milli-Q system (Millipore SAS, Molsheim, France). It contained glucose (2 g L^−1^), xylose (5 g L^−1^), furfural (3 g L^−1^), acetic acid (5 g L^−1^), and sulfates (2 g L^−1^). Table 1 shows the key physical properties from the membrane separation point of view for the compounds in the model solution. D(+)-xylose, D(+)-glucose, acetic acid glacial 100%, and sulfuric acid 95–97% were purchased from Merck KGaA, Darmstadt, Germany. Furfural (2-Furaldehyde, 99%) was purchased from Acros Organics, Geel, Belgium. The pH value of the model solution was below pH 3 and it was raised to the desired levels by adding NaOH.

The molecular weights of xylose and glucose are greater than that of furfural and acetic acid. Furthermore, the stokes diameters of these monosaccharides are higher than furfural and acetic acid. Therefore, it is expected that monosaccharides are to be rejected by NF while rejecting lower amounts of furfural and acetic acid due to their lower molecular weight and smaller diameter.

The diffusion coefficients of monosaccharides are smaller in comparison to furfural and acetic acid. The molecular dimensions of xylose and glucose are significantly higher than those of other compounds expressed by the stokes diameters, which means that the separation of the compounds from xylose and glucose can be likely achieved by membrane filtration.

Size exclusion is the dominating factor for the retention of uncharged molecules and electrostatic repulsion is the dominating factor for the retention of charged molecules [22,28]. Since the dissociation constant (pK_a_) of monosaccharides and furfural is greater than 12, they are in the uncharged state at lower pH range. In contrast, acetic acid and sulfuric acid have lower pK_a_ of 4.756, and −3, respectively, indicating that the change of pH in acidic range would significantly influence the degree of dissociation.

### 2.2. Studied Membranes

Four commercially available NF membranes obtained from two manufacturers (DuPont FilmTec, formerly Dow FilmTec (Tarragona, Spain), and Alfa Laval (Lund, Sweden)) were used in this study. Table 2 summarizes the typical membrane characteristics achieved from the manufacturer.

### 2.3. Filtration Procedure

The laboratory-scale batch membrane filtration unit CELFA P-28 (CM-Celfa Membrantrenntechnik AG, Seewen, Switzerland) is used in this study. It consists of a membrane cell, a feed tank with an external thermostat (Ecoline Staredition E100, Lauda, Germany), a circulation pump (Scherzinger 3000 3B, Furtwangen, Germany), pressure and temperature indicators, safety and discharge valves, and a nitrogen gas supply with a pressure control valve.

The crossflow circulation was kept constant at 0.47 L min^−1^ (at the circulation pump frequency of 40 Hz). The initial volume of the feed tank was 500 cm^3^ for all experiments. Circular flat sheet membranes of 75 mm in diameter with an effective surface area of 28 cm^2^ were used. The apparatus was pressurized by feeding nitrogen gas to the feed tank and the experiments were carried out at 20 bar operating pressure. The temperature of the feed solution was maintained by circulating cooling/heating water around the feed tank using an external thermostat. Temperature polarization effects were negligible due to the relatively low permeate flux through the membrane and the thermal isolation of the membrane unit.

A fresh membrane was used in each experiment and prior to use, it was compacted (initial cleaning of the membrane) inside the membrane cell, according to the manufacturer’s instructions. To determine the interaction between the solution and the membrane, water flux tests were carried out before and after each experiment. The feed, permeate, and retentate samples (2 mL each) were drawn after conditioning the membrane by permeating the feed solution until the steady state was attained (after 30 min). The concentrations of the solutes were measured with capillary electrophoresis (CE). To identify the behavior of flux reduction during the experiments, the permeate flux was measured at desired time intervals during the experiments.

### 2.4. Analytical Methods

The CE analyses were carried out with a P/ACE MDQ CE instrument (Beckman-Coulter, Fullerton, CA, USA) equipped with a diode array detector at the wavelength of 270 nm with a bandwidth of 10 nm. All samples were measured as duplicates (*n* = 2). All samples were kept in a freezer until analyzed, and they were filtered with a 0.45 µm GHP Acrodisc syringe filter (Pall Corporation, Port Washington, NY, USA) before analysis and diluted with water. Several dilution factors have been used because the concentrations of the analytes varied. The analytes were identified based on their migration times.

A modified method of Rovio et al. [29], using an uncoated fused-silica capillary of the inner diameter (I.D.) of 25 μm and the length of 30/40 cm (effective length/total length) was used for analyzing furfural, glucose, and xylose. The samples were injected at a pressure of 0.5 psi for 10 s. The used separation voltage was +16 kV and the separation time 15 min. The samples were quantified with the calibration curves of furfural, glucose, and xylose made with standard solutions. The uncoated fused-silica capillary (I.D. 75 μm and length 50/60 cm) and CEofix Anions 8 buffer kit (Analis, Namur, Belgium) were used for analyzing sulfates and acetic acid. Samples were injected at a pressure of 0.5 psi for 5 s. The used separation voltage was 30 kV and the separation time was 7 min. The samples were quantified with the calibration curves of sulfates and acetic acid made with standard solutions.

Design of Experiments (DOE) software, MODDE 12.1 (Sartorius AG, Goettingen, Germany) was used for making the experimental designs and for the data analysis.

### 2.5. Data Processing

The solute retention of a membrane, *R*, is determined as a percentage according to Equation (1):

(1) $$R\;\left(\%\right)=\left(1-\frac{C_{p}}{C_{f}}\right)\times 100$$

where *R, C_p_*, and *C_f_* represent the percentage of solute retention, the concentration of solute in the permeate, and the concentration of solute in the feed, respectively.

The permeate flux of a membrane, *J*, is calculated by Equation (2):

(2) $$J=\frac{V}{At}$$

where *J*, *V*, *A*, and *t* are the permeate flux in dm^3^ m^−2^ h^−1^, the volume of the permeate in dm^3^, the effective membrane surface area in m^2^, and the filtration time in hours, respectively.

The volumetric concentration factor (*VCF*) is the ratio of the initial volume to the final volume, and it is expressed by Equation (3):

(3) $$VCF=\frac{V_{0}}{V_{f}}$$

where *V*_0_ is the initial feed volume and *V_f_* is the final retentate volume remained in the feed tank. This is a relevant factor in experimental membrane tests with a batch operation.

The diafiltration factor (*D*) is the ratio of the consumed diafiltration diluent to the treated feed in the constant volume diafiltration, and it is determined by Equation (4):

(4) $$D=\frac{V_{d}}{V_{i}}$$

where *V_d_* is the volume of the consumed diluent and *V_i_* is the volume of the process feed stream in the feed tank.

## 3. Results and Discussion

### 3.1. Screening of Membranes

**Figure 1:**
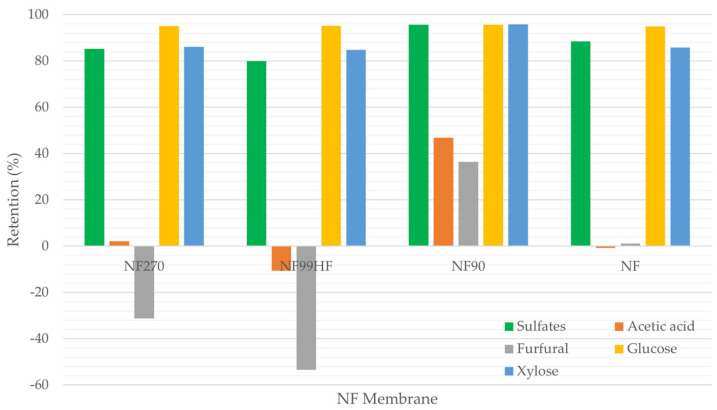
Retention of studied compounds by different nanofiltration (NF) membranes.

NF membranes were screened to determine the best performing membrane in terms of recovering the highest amounts of glucose and xylose while rejecting the maximum amounts of sulfates and acetic acid. The experiments were conducted at constant conditions, i.e., pH 3 and 25 °C with the model solution. Figure 1 shows the retention percentages of sulfates, acetic acid, furfural, glucose, and xylose for the studied NF membranes.

The retentions of xylose, glucose, and sulfates were more than 80% by all the studied membranes while exhibiting the varying retentions of acetic acid and furfural. The retentions of glucose by all the studied membranes were almost similar, with the values close to 95%, whereas the retentions of xylose were in the range of 85% to 96%. NF 90 showed the highest xylose retention compared to other membranes. However, sulfates and monosaccharides cannot be separated by NF since most of the sulfates were retained by these membranes. NF99HF had the lowest sulfates retention which is 80% and next to it, NF270 showed 85% retention. Acetic acid and furfural retentions by NF90 (47% and 36%, respectively) were significantly higher than those of the other membranes. As per Figure 1, significant negative retentions were observed for furfural by NF270 and NF99HF, and for acetic acid by NF99HF, which indicate that acetic acid and furfural were enriched in the permeate compared with the feed concentration. Therefore, the negative retention of acetic acid and furfural is favorable to permeate them rapidly from the stream. The retention percentages of acetic acid and furfural by NF and acetic acid by NF270 were close to each other.

Bearing in mind the high retentions for xylose (85%) and glucose (95%), NF99HF appears to be the most promising membrane to concentrate monosaccharides with the minimum retention of sulfates (80%) out of the studied membranes. For acetic acid and furfural, higher negative retentions were observed for NF99HF in comparison to the negative retentions by NF270, which means more permeate enrichment of acetic acid and furfural by NF99HF.

Nguyen et al. [21] and Qi et al. [13] studied the detoxification of monosaccharides by NF90 and NF270 membranes with model solutions containing glucose, xylose, and furfural. In comparison to their results, somewhat similar retentions for glucose and xylose were obtained in our findings even though their model solution did not contain sulfates. However, the retention of furfural is significantly varied in our studies this being possibly the effect of sulfates existing in our model solution.

In a nutshell, NF99HF was the most favored membrane to concentrate monosaccharides with the minimum retention of sulfates, while NF90 was found to retain all compounds, and therefore being plausible for purifying process waters.

### 3.2. Effect of Sulfates

**Figure 2:**
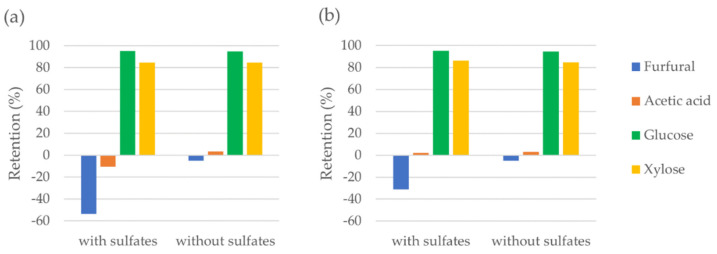
Effect of sulfates on the negative retention with (**a**) NF99HF and (**b**) NF270.

The observed negative retention of furfural and acetic acid possibly occurs due to the interaction between sulfates and other compounds in the feed solution. Sulfuric acid is used for the pretreatment of biomass and sulfates remain in the filtrate at the end of processes. For instance, in the organosolv process, sulfuric acid is added to the pulping liquor, and sulfates remain in the filtrate even after the lignin precipitation and solvent recovery. Since it is important to investigate the effect of sulfates on the negative retention, the NF99HF and NF270 membranes were studied with and without sulfates in the feed stream. The model solution was used for the experiments with sulfates. A model solution without sulfates, while keeping other remaining compound concentrations unchanged, was prepared, and used for the experiments not including sulfates. Figure 2 shows the effect of sulfates on the negative retention of furfural and acetic acid by NF99HF and NF270 along with the retention of glucose and xylose.

As per Figure 2, the retentions of glucose and xylose were independent of the sulfates content in the stream. However, the effect of sulfates on the negative retentions of furfural and acetic acid can clearly be seen. Both NF99HF and NF270 showed more negative retentions with sulfates than without sulfates in the stream. This result showed that the presence of sulfates enhances negative retention of acetic acid and furfural possibly due to the intermolecular interactions in the feed occurring by sulfates. It can be explained as follows, the presence of sulfates having high retention induces an increased passage of acetic acid and furfural through the membrane due to the increased electric potential. The study of Schlackl et al. [17] suggests a similar explanation for the observed phenomena. This effect could be studied in more detail by changing the concentration of sulfates and with the presence of other salts.

To summarize, sulfates cannot be separated from monosaccharides effectively by NF and the presence of sulfates favors the separation of acetic acid from monosaccharides.

### 3.3. Effect of pH and Temperature on Solute Retention

The effect of pH and temperature was investigated with the identified best performing membrane, NF99HF. The aim of this study is to understand the effects of pH and temperature on the monosaccharide recovery and purity of the concentrated monosaccharides.

The range of pH was selected based on the recommended operating pH range of the membrane, which is pH 3–10 for NF99HF. pH 3 was chosen as the minimum pH value since it is the minimum recommended pH for the membrane. From the literature, it is expected that low pH is more favorable for the separation [22]. The range of temperature was selected based on the maximum operating temperature of the membrane, which is 50 °C for NF99HF. It should be noticed that the realistic temperatures of the comparable biorefinery streams can exceed the maximum operating temperature of the membrane, being ~75 °C, for example the temperature of the Fraunhofer organosolv stream after the solvent recovery. Thus, the temperature might need to be lowered before the membrane operation in real biorefinery applications. For this study, 45 °C was selected as the highest temperature while choosing the room temperature (25 °C) as the lowest.

When studying the effects of pH and temperature on the retention of each solute, a full factorial (3 level) design having 11 experiments was performed. The quantitative temperature factor with low (25 °C) and high (45 °C) and the quantitative multilevel pH factor with pH 3, 5, and 7 were used. The experimental matrix designed by the analytical software MODDE, is shown in Table 3 with the retention percentages obtained by the experiments. Experiment 5 was excluded as it deviates from other two center points and was highlighted as a possible outlier in the MODDE software. The model was fitted with multiple linear regression (MLR) and validity of the model was tested with the analysis of variance (ANOVA) with the confidence level of 95%. The general form of the model equation for the measured output variables was quadratic as follows:

(5) $$y_{i}=b_{0}+b_{1}x_{1}+b_{2}x_{2}+b_{3}x_{1}x_{2}+b_{4}x_{1}^{2}+b_{5}x_{2}^{2}$$

Here $y_{i}$ is the output variable as interpreted in Table 4, $b_{0}$, $b_{1}$, $b_{2}$, $b_{3}$, $b_{4}$, and $b_{5}$ are the regression coefficients, and $x_{1}$ and $x_{2}$ are the input variables pH and temperature, respectively. The coefficients of the models identified are given in Table 4. Table 5 shows the summary of results for the models derived from the MODDE analysis.

As shown in Table 5, R^2^ indicates that the models fit to the experimental data very well. In addition, our models are significant according to the Q^2^ values being greater than 0.1. The Model validity for all variables indicates that there are no significant model problems and the Reproducibility values show as well that our models have high reproducibility. As per the ANOVA test, the probabilities (*p*) of the regressions are significant at 95% confidence level and thus, the models are statistically good. The probabilities for the lack of fit values are not significant at 95% confidence level and hence, statistically, the modes have no lack of fit. Therefore, all the models could be used to predict the response function accurately.

**Figure 3:**
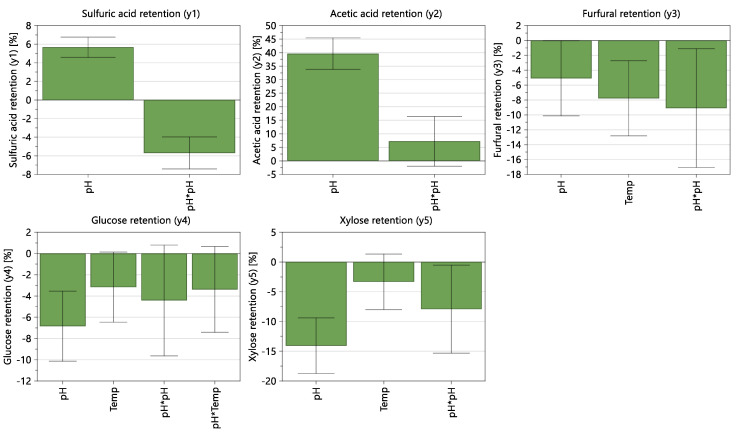
Coefficient plots for the identified models (scaled and centered).

Figure 3 shows the coefficient plots displaying the regression coefficients with confidence intervals for the identified models. Coefficient plots for each model were obtained after removing insignificant and small effects from the model. The effect of temperature is less significant than the effect of pH. pH and the quadratic term of “pH * pH” have significant effects on all retentions whereas pH has the highest effect on solute retentions, except furfural. The quadratic term “Temp * Temp” has an insignificant effect on all retention values. There is no significant temperature effect or interaction effect of temperature on the retentions of sulfuric acid and acetic acid.

**Figure 4:**
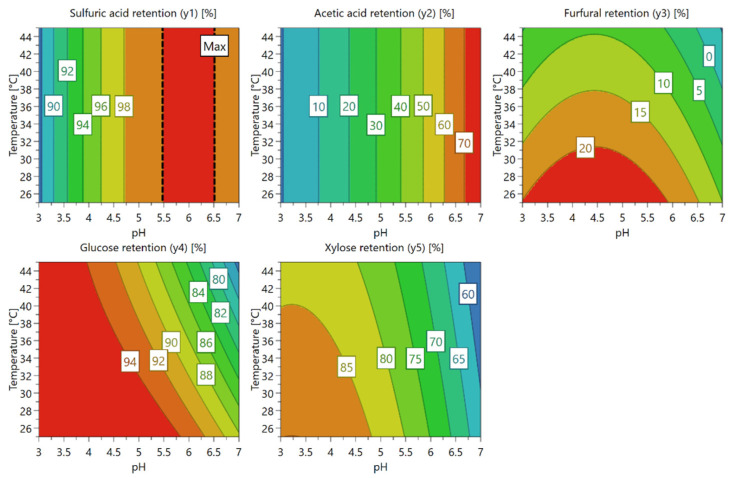
Response contour plots for each response.

Figure 4 shows the response contour plots displaying the predicted response values for each response, spanned by two factors (pH and temperature). The response contour plots show that the temperature is considerably less significant to the retention values than pH, except for the furfural retention. For sulfuric acid and acetic acid, there is no significant temperature effect, and their retentions completely depend on the pH. Sulfuric acid and acetic acid retentions are minimized in lower pH values even though the sulfuric retention value is considerably high (about 88% in pH 3). Qi et al. [13] showed that the retentions of monosaccharides and furfural decreased with increasing pH, which is in line with our findings for the effect of pH. With the studied model solution, the projected retentions of glucose, xylose, furfural, sulfates, and acetic acid by NF99HF are 95%, 90%, 20%, 88%, and 0%, respectively at pH 3 and temperature of 25 °C.

Ultimately, lower pH and lower temperature conditions are favored to maximize the monosaccharide retentions and to remove acetic acid. In addition, a higher furfural retention value can be seen in those conditions.

### 3.4. Effect of pH and Temperature on Membrane Fouling

**Figure 5:**
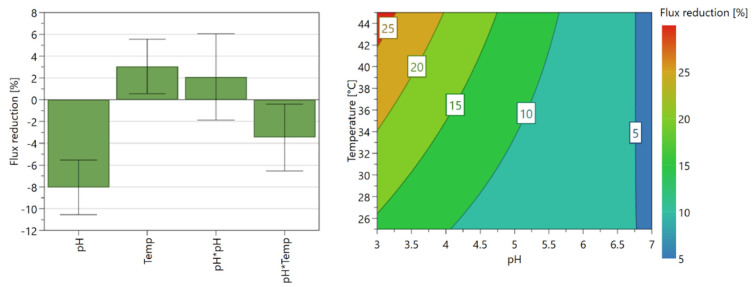
Coefficient plot and response contour plot for pure water flux reductions.

The pure water fluxes were measured before and after each experiment was conducted to study the pH and temperature effects, and to identify the fouling tendency. Table 6 shows the permeate flux values and the flux reduction percentages. The water flux measurement before the experiment was taken after the membrane compaction and the water flux after the experiment was measured after cleaning the membrane. The membrane was cleaned for about 30 min circulating pure water under pressure of 2 bar and temperature of 30 °C inside the membrane cell. Figure 5 shows the coefficient plot and the response contour plot of pure water flux reductions.

The results show a significant fouling tendency in the NF and the higher flux reduction indicates the higher fouling effect. As per Figure 5, pH is the factor affecting most the fouling tendency and the temperature has also a significant effect on that. At lower pH and higher temperatures, the fouling tendency is maximized. However, as per the results obtained for the effect of pH and temperature, the NF must be performed at lower pH and lower temperatures to obtain the expected separation efficiency.

The membranes did not undergo any chemical cleaning after the experiments which could have overcome irreversible fouling to a certain extent [3]. Nevertheless, the real biorefinery HMC streams will probably lead to more significant fouling since more particles, and ashes exist in the streams. The selection of operating pressure, crossflow velocity and feed concentration will also affect the fouling tendency and these parameters will be studied in detail in the next phase.

In summary, the fouling tendency is minimized at high pH and low temperatures.

### 3.5. Role of Diafiltration

Diafiltration is a particular type of NF process in which the retentate of the primary NF stage is diluted with a diluent and filtered in another NF stage. This aims to enhance the removal degree of permeate compounds that are partly retained by the NF stage and thus, helps to obtain high purity products. Typically, diafiltration uses the same membrane type as the one used in the NF stage.

In this study, diafiltration can be used to purify and concentrate the retained monosaccharides by reducing the concentration of permeated compounds. The monosaccharides containing impurities is fed to the primary NF stage and there, it is expected to separate the monosaccharides and impurities partially (more monosaccharides with lower amount of impurities in the retentate). The retentate from the primary NF stage with the addition of diafiltration diluent, in this case Milli-Q water, is directed to the diafiltration stage. The retentate obtained from the diafiltration stage has more purified monosaccharides. Depending on the required purity, *D* (see Equation (4)) can be chosen.

**Figure 6:**
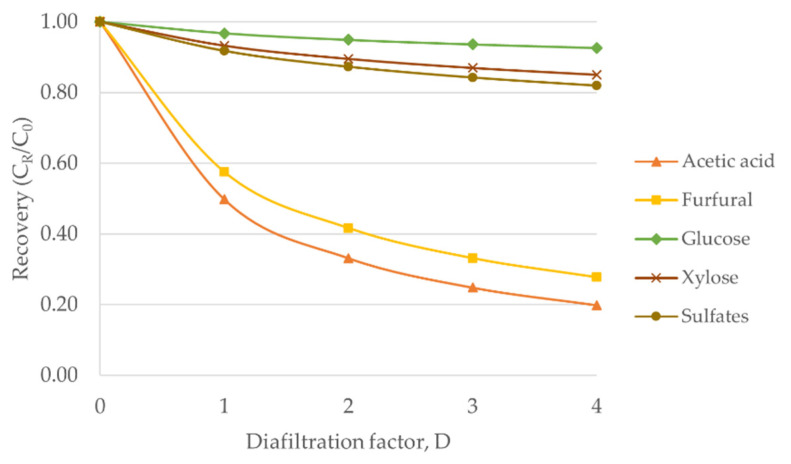
Estimated effects of diafiltration by NF99HF membrane.

The estimations for the effect of diafiltration were formulated and are presented in Figure 6. It demonstrates the estimated recovery of each compound with respect to *D* by the NF99HF membrane. *C_R_* is the concentration of retentate in diafiltration stage, whereas *C*_0_ is the concentration of retentate in the primary NF stage. The estimations are based on results obtained in the studied experimental model at the optimized conditions (pH 3 and 25 °C).

Based on the diafiltration estimations in Figure 6, it is expected that 67% removal of acetic acid at *D* = 2 is obtained while losing 5% of glucose and 10% of xylose. When increasing *D* to 4, it is estimated that the removal of acetic acid is 80% whereas at the same time, slightly more monosaccharides are lost, and an excessive amount of water is consumed. When assuming *VCF* = 5, purity of 73% in the concentrated monosaccharides is predicted to be achieved by NF and diafiltration with *D* = 2 for the studied model solution. If the *VCF* value is assumed to be 10, then the purity becomes 76%, but this means much higher energy demand because of the need of increased filtration capacity.

In brief, diafiltration can be used to lower the concentrations of acetic acid and furfural, thereby enhancing the purity of the monosaccharides.

## 4. Conclusions

The concentration and purification of monosaccharides were studied by four NF membranes with a model solution containing glucose, xylose, furfural, acetic acid, and sulfates. The results of the investigation show that:

- NF99HF was the best performing membrane to concentrate the monosaccharides, with the high retentions of 85% for xylose and 95% for glucose and with the minimum retention of sulfates.
- pH has the highest effect on the retentions of the studied solutes and no significant effect of temperature was observed on the retention of sulfates and acetic acid.
- Lower pH and lower temperature conditions are desired to maximize the retention of monosaccharides and to maximize the removal of acetic acid while retaining more furfural with the monosaccharides.
- Sulfates cannot be separated from monosaccharides effectively by NF and most of the sulfates remain with the monosaccharides.
- Diafiltration is used to enhance the purity of the concentrated monosaccharides. Theoretically, 73% purity of monosaccharides is expected with *VCF* = 5 and *D* = 2 for the studied model solution.
- The effect of sulfates on the solute retention was investigated and the results indicated that the presence of sulfates favors the separation of acetic acid and furfural from monosaccharides.
- Fouling tendency is maximized at low pH and high temperatures. However, the model solution used can give only a limited insight to this phenomenon and thus, a systematic evaluation of the fouling tendency will be conducted in the future.

## Figures and Tables

**Table 1:** Physical properties of compounds in the model solution.

Compound	Molecular Formula	Molecular Weight (g mol^−1^)	Dissociation Constant (pK_a_)	Stokes Diameter (nm)	Diffusion Coefficient at 25 °C (10^−6^ cm^2^ s^−1^)
Glucose	C_6_H_12_O_6_	180.16	12.28 [24]	0.726 [25]	6.76 [26]
Xylose	C_5_H_10_O_5_	150.13	12.15 [24]	0.638 [25]	7.69 [26]
Furfural	C_5_H_14_O_2_	96.08	>12 [24]	0.438 [25]	11.2 [25]
Acetic acid	CH_3_COOH	60.05	4.756 [24]	0.412 [25]	11.9 [25]
Sulfuric acid	H_2_SO_4_	98.08	−3 [27]	-	-

**Table 2:** Characteristics of the studied membranes.

	NF90	NF270	NF	NF99HF
Manufacturer	DuPont FilmTec	DuPont FilmTec	Alfa Laval	Alfa Laval
Support material	Polysulfone	Polysulfone	Polyester	Polyester
Type	Polyamide thinfilm composite	Polypiperazine thinfilm composite	Thinfilm composite	Thinfilm composite
Rejection	>97% MgSO_4_	>97% MgSO_4_	≥99% MgSO_4_	≥99% MgSO_4_
pH range	2–11	3–10	3–10	3–10
Maximum pressure (bar)	41	41	55	55
Maximum temperature (°C)	45	45	50	50

**Table 3:** Experimental matrix for factor studies and retention percentages by NF99HF.

Exp No.	Factors		Responses				
	pH	Temp (°C)	$y_{1}$ (%)	$y_{2}$ (%)	$y_{3}$ (%)	$y_{4}$ (%)	$y_{5}$ (%)
1	3	25	89.65	−3.18	21.85	97.15	92.14
2	5	25	97.75	25.04	21.06	92.71	78.95
3	7	25	99.27	81.81	16.36	89.00	61.34
4	3	35	86.99	−3.80	11.45	95.15	87.12
5	5	35	99.28	14.54	−4.80	93.74	74.47
6	7	35	98.96	83.52	−6.91	83.95	64.39
7	3	45	86.17	5.25	4.16	94.10	80.59
8	5	45	99.48	39.28	11.02	93.34	82.15
9	7	45	98.60	70.79	−2.53	72.41	49.72
10	5	35	99.20	33.25	16.53	91.37	78.70
11	5	35	99.46	29.79	17.35	94.76	82.09

**Table 4:** The coefficient list of the identified models.

Output	Output Variable	$b_{0}$	$b_{1}$	$b_{2}$	$b_{3}$	$b_{4}$	$b_{5}$
$y_{1}$	Sulfuric acid retention (%)	98.973	5.670	-	-	−5.699	-
$y_{2}$	Acetic acid retention (%)	31.840	39.642	-	-	7.225	-
$y_{3}$	Furfural retention (%)	16.490	−5.090	−7.770	-	−9.093	-
$y_{4}$	Glucose retention (%)	93.045	−6.840	−3.168	−3.385	−4.418	-
$y_{5}$	Xylose retention (%)	80.473	−14.067	−7.923	-	−3.328	-

**Table 5:** Summary of results for the identified models.

Output Variable	R^2^	Q^2^	Model Validity	Reproducibility	Regression *p*	Lack of Fit *p*
Sulfuric acid retention (%)	0.968	0.932	0.459	0.999	0.000	0.115
Acetic acid retention (%)	0.974	0.947	0.684	0.994	0.000	0.283
Furfural retention (%)	0.824	0.489	0.366	0.997	0.011	0.079
Glucose retention (%)	0.898	0.328	0.822	0.893	0.011	0.492
Xylose retention (%)	0.914	0.740	0.735	0.966	0.001	0.347

**Table 6:** Pure water flux measurements before and after experiments.

Exp No.	pH	Temp (°C)	Pure Water Flux, *J* (dm^3^ m^−2^ h^−1^)		d
			Before	After	
1	3	25	358	301	16.09
2	5	25	340	316	7.12
3	7	25	372	356	4.43
4	3	35	329	274	16.74
5	5	35	345	305	11.73
6	7	35	350	330	5.69
7	3	45	372	265	28.94
8	5	45	367	317	13.59
9	7	45	341	329	3.40
10	5	35	366	322	12.00
11	5	35	356	323	9.16

## Data Availability

The data presented in this study are available within the article.
